# The Relationship Between Childhood Trauma and Shame: The Mediating Role of Dissociation

**DOI:** 10.3390/ejihpe15080151

**Published:** 2025-08-07

**Authors:** Gianluca Santoro, Lucia Sideli, Alessandro Musetti, Adriano Schimmenti

**Affiliations:** 1 Department of Humanities, Social Sciences and Cultural Industries, University of Parma, Borgo Carissimi 10, 43121 Parma, Italy; alessandro.musetti@unipr.it; 2 Department of Human Sciences, LUMSA University, Piazza delle Vaschette 101, 00193 Rome, Italy; l.sideli@lumsa.it; 3 Department of Human and Social Sciences, UKE—Kore University of Enna, Piazza dell’Università, 94100 Enna, Italy; adriano.schimmenti@unikore.it

**Keywords:** childhood trauma, child abuse, child neglect, dissociation, psychodynamics, shame, structural equation modeling

## Abstract

Previous research has found significant associations among childhood trauma, dissociation, and shame. Furthermore, the clinical literature suggests that dissociation may foster feelings of shame in individuals who were exposed to childhood trauma. The current study aimed to test the potential mediating effect of dissociation on the association between childhood trauma and shame. The study sample consisted of 763 adults (479 females, 62.8%) from the general Italian population, aged between 18 and 65 years (M = 31.31, SD = 13.29). Self-report instruments assessing childhood trauma, dissociation, and shame were administered to participants via an anonymous online survey. Structural equation modeling showed that childhood trauma was associated with increased levels of both dissociation and shame. Moreover, dissociation partially mediated the predictive association between childhood trauma and shame. These findings suggest that dissociation might heighten the tendency to unconsciously reenact self-devaluation and self-blame in individuals exposed to childhood trauma, increasing feelings of shame.

## 1. Introduction

Childhood trauma encompasses a range of adverse experiences, including various forms of abuse and neglect, which reflect significant failures in caregiving. Specifically, child abuse refers to acts of commission—deliberate actions that cause harm—whereas child neglect involves acts of omission, or the failure to provide necessary care and protection (McCoy & Keen, 2022). Childhood traumatic experiences may include emotional, physical, and sexual abuse, as well as emotional and physical neglect (Bernstein et al., 2003). The detrimental impact of childhood trauma on psychological development has been widely documented, with research consistently highlighting its profound effects on bodily and self-perception, affect regulation, and relational functioning (Marusak et al., 2015; Riazi & Manouchehri, 2024; Schmitz et al., 2023; Van Der Kolk et al., 2005; Watts et al., 2023). Central to this impact is the violation of trust and safety that trauma often entails. When caregivers, who are supposed to provide protection and validation, instead inflict harm or fail to meet basic emotional needs, children may internalize these experiences as evidence of personal defectiveness. This internalization fosters a pervasive sense of shame, rooted in the belief that one is fundamentally flawed, unworthy, or unlovable (Schimmenti, 2012).

Shame is a deeply ingrained self-conscious emotion that arises from a perceived failure to meet social or personal standards, often leading to feelings of unworthiness and self-contempt (M. Lewis, 1992). Unlike guilt, which pertains to specific actions, shame involves a global negative evaluation of the self. Individuals experiencing shame may feel that their flaws are potentially exposed not only to themselves but also to others, regardless of whether anyone is actually present (H. B. Lewis, 1971; Miller & Tangney, 1994; Tangney, 2001). Notably, shame is shaped by early relational experiences and typically serves as a regulatory mechanism for social belonging (Herman, 2012; Schore, 1998). Under optimal developmental conditions, it serves an adaptive role by reinforcing interpersonal bonds and guiding moral behavior. However, in the context of childhood trauma, shame often becomes maladaptive. Individuals with a history of childhood trauma may experience unprocessed feelings of shame which entail a pervasive sense of inferiority and unworthiness. In such circumstances, shame no longer regulates social behavior constructively but instead fosters chronic self-criticism, social withdrawal, and in some cases pathological personality functioning (Benau, 2017; Schimmenti, 2012). Accordingly, the tendency to experience shame is one of the problematic consequences of child abuse and neglect (Flach & Cariola, 2025; L. Zhang et al., 2023), as further evidenced by research indicating that shame is associated with elevated levels of psychopathology (e.g., DeCou et al., 2023; Nechita et al., 2021; Swee et al., 2021). Previous research suggests that various types of childhood trauma may contribute to heightened levels of shame (Archuleta et al., 2024; Dorahy & Clearwater, 2012; Keene & Epps, 2016; O’Loghlen et al., 2023; Ross et al., 2019). Furthermore, recent meta-analytic findings showed that overall childhood trauma, as well as specific childhood traumatic experiences such as emotional abuse, sexual abuse, emotional neglect, and physical neglect, is positively associated with shame (H. Zhang et al., 2025). Additionally, it has been found that certain psychological factors may mediate the association between childhood trauma and shame. Talmon and Ginzburg (2017) showed that self-objectification and shame, as well as a sense of disrupted body boundaries and shame, serially mediated the relationship between childhood trauma and decreased levels of well-being in a sample of female college and university students. Mojallal et al. (2021) found that the early maladaptive schemas concerning incompetence mediated the positive association between child neglect and shame-related withdrawal. These findings suggest that shame may emerge through complex psychological pathways shaped by early traumatic experiences. Nonetheless, other potential psychological factors might explain the relationship between childhood trauma and shame.

The American Psychiatric Association (2022) classifies dissociative disorders as conditions characterized by a disruption in the integration of various domains of psychological functioning, including consciousness, memory, identity, emotion, perception, body representation, motor control, and behavior. From a psychodynamic perspective, these disorders are understood as the outcome of persistent dissociative processes. Dissociation itself is conceptualized as a common psychological mechanism that allows individuals to cope with distressing experiences by splitting off emotions, sensations, or memories from awareness. While this mechanism can serve an adaptive function in facing acute stress (e.g., a temporary sense of disconnection from oneself or environment during threatening events), chronic dissociation, particularly in survivors of childhood trauma, may disrupt the integration of self-experiences. This can result in identity fragmentation and difficulties in emotion regulation (Bromberg, 1998, 2011; R. A. Chefetz, 2015; Santoro et al., 2025). In such cases, dissociation often represents an attempt to preserve a sense of emotional safety and self-coherence by compartmentalizing experiences that are too painful to process consciously (Schimmenti & Caretti, 2016). When rooted in attachment trauma, this form of pathological dissociation typically emerges as a defensive response to a paradoxical relational dynamic in which the caregiver, who should function as a source of safety, is simultaneously the source of fear. This creates a state of relational disorganization, forcing the child into a psychological impasse (Main & Hesse, 1990). To face this conflict, dissociative processes may activate a form of psychological splitting, whereby painful aspects of the self, experiences, and relationships are sequestered into segregated mental states (Bowlby, 1980). While this mechanism may offer temporary protection from acute distress, it often leads to long-term disruptions in identity cohesion and emotion regulation (Schimmenti, 2023). There is consistent evidence that child abuse and neglect are linked to heightened dissociation (Haferkamp et al., 2015; Kate et al., 2021; Simeon & Putnam, 2022; Vonderlin et al., 2018).

Meta-analytic findings revealed a moderate positive association between dissociation and shame (Rudy et al., 2022). Additionally, research suggests a mutual relationship between dissociation and shame. Some studies found that induced dissociative experiences lead to increased feelings of shame (Dorahy et al., 2021; Platt et al., 2017), whereas other studies showed significant effects of evoking-shame narrative scripts and shame-related memories in enhancing dissociative experiences (Dorahy et al., 2017; Kouri et al., 2023).

Schimmenti (2022) proposed a dynamic relationship between chronic dissociation and shame among victims of childhood abuse and neglect. Dissociation allows individuals to temporarily split-off overwhelming affective states, but at the same time it paradoxically reinforces fragmented and shame-laden self-representations. Specifically, the dissociative compartmentalization of traumatic experiences impedes their integration into a coherent self-narrative, often leading individuals to unconsciously reenact patterns of self-devaluation and self-blame (Costanzo et al., 2023). In this sense, dissociation is not merely a transient response to acute stress but a persistent mechanism that reinforces negative self-representations and sustains a cycle of chronic shame (Bromberg, 1998). Accordingly, shame represents an organizing principle of identity among individuals who were exposed to child abuse and neglect (Schimmenti, 2022).

In line with these theoretical advancements, dissociation may serve as a key explanatory mechanism of the relationship between childhood trauma and shame. By investigating the mediating role of dissociation in the relationship between childhood trauma and shame, this study aims to contribute to a deeper understanding of trauma’s lasting imprint on maltreated children. Specifically, we predicted that (a) childhood trauma is associated with increased levels of both dissociation and shame, (b) dissociation is associated with increased levels of shame, and (c) dissociation has a mediating effect on the relationship between childhood trauma and shame.

## 2. Materials and Methods

### 2.1. Participants and Procedures

The study sample comprised 763 adults from the general Italian population (479 females, 62.8%). Participants ranged in age from 18 to 65 years (M = 31.31, SD = 13.29) and reported a mean of 14.35 years of education (SD = 2.77). Approximately one quarter of participants (n = 193, 25.3%) reported being married. Almost half of the participants (n = 380, 49.8%) were students, 32.4% (n = 247) were employed, 8.3% (n = 63) were self-employed, 5.9% (n = 45) were unemployed, 2.8% (n = 21) were homemakers, and 0.9% (n = 7) were retired. There were no significant sex differences in age (*t*_(761)_ = 1.30, *p* = 0.193), years of education (*t*_(761)_ = −0.34; *p* = 0.738), and marital status (χ^2^_(1)_ = 1.01, *p* = 0.315).

A snowball sampling procedure was used to recruit participants. Various announcements in Italian were posted on social media platforms (e.g., Facebook and WhatsApp). Each announcement presented a link to an anonymous online survey, which included an informed consent schedule and self-report instruments. Individuals who consented to participate in the study were automatically directed to complete the self-report instruments. The announcements also contained a request for recipients to disseminate the online survey link to others. All participants were volunteers and received no compensation. The following inclusion criteria were applied: (a) being between 18 and 65 years of age, and (b) being a native or fluent Italian speaker. No exclusion criteria were established. The present study was designed as part of a larger research project, the dataset of which had not been previously used in any other studies. All data were collected between April 2021 and March 2022. The current study was conducted in accordance with the Declaration of Helsinki, and its procedures were approved by the IRB for psychological research of the UKE—Kore University of Enna (protocol code UKE-IRBPSY-04.21.03, 13 April 2021).

### 2.2. Measures

The Experience of Shame Scale (ESS; Andrews et al., 2002; Italian validation by Velotti et al., 2017) is a self-report instrument that assesses shame feelings. The ESS consists of 25 items that are ranked on a 4-point Likert scale (1 = “Not at all”; 4 = “Very much”). It includes a total scale and three subscales that evaluate characterological shame (i.e., shame about one’s own psychological characteristics; 12 items), behavioral shame (i.e., shame about one’s own behavior; 9 items), and bodily shame (i.e., shame about one’s own body or specific body parts; 4 items), respectively. Higher scores indicate higher levels of shame. Examples of items are: “Have you worried about what other people think of the sort of person you are?” (related to characterological shame); “Have you worried about what other people think of you when you do something wrong?” (related to behavioral shame); “Have you worried about what other people think of your appearance?” (related to bodily shame). The ESS demonstrated construct and discriminant validity (Andrews et al., 2002), as well as structural validity and test–retest reliability in both clinical and non-clinical samples (Vizin et al., 2016). In the current study, Cronbach’s alpha was 0.96 for the total scale, 0.93 for characterological shame, 0.91 for behavioral shame, and 0.89 for bodily shame.

The Childhood Trauma Questionnaire–Short Form (CTQ-SF; Bernstein et al., 2003; Italian validation by Sacchi et al., 2018) is a self-report instrument that assesses five types of childhood traumatic experiences, such as emotional abuse, physical abuse, sexual abuse, emotional neglect, and physical neglect. The CTQ-SF comprises 28 items that are rated on a 5-point Likert scale (1 = “Never true”, 5 = “Very often true”). Each type of traumatic experience is evaluated through 5 items. Higher scores indicate greater exposure to childhood traumatic experiences. Three items evaluate the degree of minimization or denial of childhood traumatic experiences. As the current study was aimed to investigate the relationships between childhood trauma, dissociation, and shame, we did not employ the minimization/denial scale. Examples of items are the following: “When I was growing up people in my family said hurtful or insulting things to me” (related to emotional abuse); “When I was growing up people in my family hit me so hard that it left me with bruises or marks” (related to physical abuse); “When I was growing up I believed that I was sexually abused” (related to sexual abuse); “When I was growing up my family was a source of strength and support” (related to emotional neglect; reverse-scored item); “When I was growing up I had to wear dirty clothes” (related to physical neglect); “When I was growing up I had the perfect childhood” (related to minimization/denial). The CTQ-SF showed adequate psychometric properties, such as structural validity, measurement invariance, and reliability (Badenes-Ribera et al., 2024; Georgieva et al., 2021). In the current study, Cronbach’s alpha was 0.84 for emotional abuse, 0.86 for physical abuse, 0.91 for sexual abuse, 0.88 for emotional neglect, and 0.63 for physical neglect.

The Dissociative Symptoms Scale (DSS; Carlson et al., 2018; Italian validation by Schimmenti et al., 2020) is a self-report instrument that assesses dissociative symptoms. The DSS comprises 20 items. Participants were asked to rate how frequently each dissociative symptom had occurred in the past week on a 5-point Likert scale (0 = “Not at all”; 4 = “More than one a day”). This instrument includes a total scale and four subscales evaluating specific types of dissociative symptoms: depersonalization/derealization (6 items), sensory misperceptions (5 items), cognitive/behavioral reexperiencing (4 items), and gaps in awareness and memory (5 items). Higher scores indicate greater severity of dissociative symptoms. The following statements are examples of items: “I felt like I was outside myself, watching myself do things” (related to depersonalization/derealization); “I smelled something that I know really wasn’t there” (related to sensory misperception); “I reacted to people or situations as if I were back in an upsetting time in my past” (related to cognitive/behavioral reexperiencing); “I couldn’t remember things that had happened during the day even when I tried to” (related to gaps in awareness and memory). The DSS demonstrated good psychometric properties, including validity and reliability (Carlson et al., 2018; Jo & Choi, 2023; Schimmenti et al., 2020). In the current study, Cronbach’s alpha was 0.94 for the total scale, 0.86 for depersonalization/derealization, 0.82 for sensory misperceptions, 0.85 for cognitive/behavioral reexperiencing, and 0.85 for gaps in awareness and memory.

A sociodemographic schedule was also employed to collect information on sex, age, years of education, marital status, and employment status.

### 2.3. Statistical Analyses

Descriptive statistics were estimated for all the variables of the study. Sex differences were examined through the *t*-test and chi-squared test. Associations between age, years of education, childhood traumatic experiences, dissociative symptoms, and feelings of shame were investigated through Pearson’s *r* correlation coefficients. A structural equation modeling (SEM) was performed to examine the direct and indirect effects of childhood trauma on shame. Specifically, the model included the following paths: (a) childhood trauma was specified as a predictor of both dissociation and shame; (b) dissociation was specified as a predictor of shame; (c) dissociation was specified as a mediating variable in the association between childhood trauma and shame. Furthermore, sociodemographic characteristics (i.e., sex, age, and years of education) were specified as covariates predicting both dissociation and shame. The model was estimated through the diagonally weighted least squares (DWLS) method, as it does not rely on distributional assumptions about the observed variables. The model included three latent variables: (a) scores on CTQ-SF scales (i.e., emotional abuse, physical abuse, sexual abuse, emotional neglect, physical neglect) were used as indicators of the latent variable childhood trauma; (b) scores on DSS subscales (i.e., depersonalization/derealization, sensory misperceptions, cognitive/behavioral reexperiencing, gaps in awareness and memory) were used as indicators of the latent variable dissociation; and (c) scores on ESS subscales (i.e., characterological shame, behavioral shame, bodily shame) were used as indicators of the latent variable shame. The values of sex, age, and years of education were entered as observed variables. The goodness of fit of the model was evaluated using the following indices: the chi-square/degrees of freedom ratio (χ^2^/df), the Comparative Fit Index (CFI), the Tucker-–Lewis Index (TLI), the Standardized Root Mean Square Residual (SRMR), and the Root Mean Square Error of Approximation (RMSEA). The model is considered to demonstrate an acceptable fit when χ^2^/df values are below 3, CFI and TLI values are above 0.95, SRMR values are below 0.08, and RMSEA values are below 0.07 (Hooper et al., 2008). The statistical significance of the indirect effect of dissociation within the model was further investigated using bootstrap analysis with 5000 units: if the 95% confidence interval (C.I.) does not contain the value of 0, the indirect effect is statistically significant at the critical level of 0.05. The R Package lavaan (version 0.6–19; Rosseel, 2012) was used to perform SEM.

## 3. Results

Descriptive statistics and sex differences are shown in Table 1. Males reported more experiences of physical abuse and neglect, whereas females reported a greater severity of dissociative symptoms—especially cognitive/behavioral reexperiencing—and shame, including characterological, behavioral, and bodily shame. Correlation analyses showed significant and positive associations among childhood traumatic experiences, dissociative symptoms and shame, with the exception of a non-significant association between physical neglect and bodily shame. Age was significantly associated with decreased levels of dissociative symptoms, including depersonalization/derealization, cognitive/behavioral reexperiencing, and gaps in awareness and memory, as well as with reduced levels of all forms of shame. Years of education were significantly associated with decreased severity of all types of dissociative symptoms and reduced levels of shame, especially characterological shame (see Table 2).

**Figure 1:**
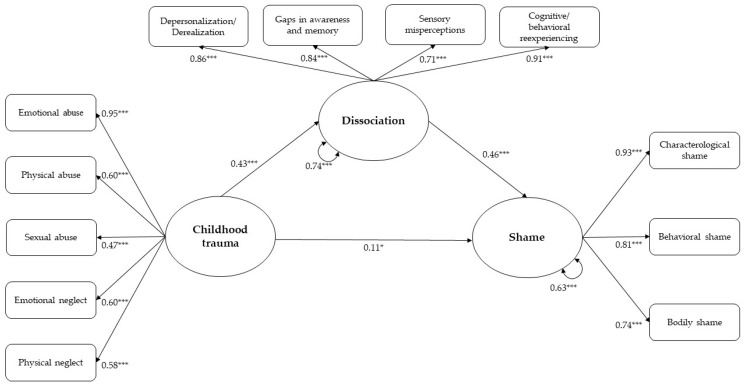
Structural equation modeling of the relationships between childhood trauma, dissociation and shame. Note: Sex, age, and years of education were significant covariates in the model; * *p* < 0.05; *** *p* < 0.001.

SEM analysis showed that the structural model included significant associations between childhood trauma, dissociation, and shame: childhood trauma was associated with heightened levels of both dissociation and shame; dissociation was associated with increased shame; dissociation was a significant mediating variable in the positive association between childhood trauma and shame. Bootstrap analyses provided further support for the mediating role of dissociation within the model. Specifically, the direct, indirect and total effects of childhood trauma on shame were significant (see Table 3). Thus, the final model showed that dissociation partially mediated the relationship between childhood trauma and shame. Additionally, sociodemographic variables emerged as significant covariates in the model: being female was associated with higher levels of both dissociation (β = 0.07; *p* = 0.041) and shame (β = 0.17; *p* < 0.001); younger age predicted heightened levels of both dissociation (β = −0.17; *p* < 0.001) and shame (β = −0.19; *p* < 0.001); and years of education were negatively associated with dissociation (β = −0.18; *p* < 0.001). The tested model demonstrated a satisfactory goodness of fit as follows: χ^2^/df = 2.699; CFI = 0.973, TLI = 0.966, SRMR = 0.059; RMSEA = 0.047 (90% C.I. [0.040–0.055]). The standardized estimates of the final model are displayed in Figure 1.

## 4. Discussion

The current study was aimed at investigating the mediating effect of dissociation on the relationship between childhood trauma and shame. Descriptive statistics indicated that the mean CTQ-SF subscale scores were 7.97 for emotional abuse, 6.01 for physical abuse, 6.13 for sexual abuse, 10.03 for emotional neglect, and 6.60 for physical neglect. These values suggest that participants reported relatively lower levels of childhood traumatic experiences compared to the global population. Indeed, previous meta-analytic findings showed that overall pooled mean estimates of CTQ subscales were equal to 9.52 for emotional abuse, 7.43 for physical abuse, 6.92 for sexual abuse, 11.34 for emotional neglect, and 7.99 for physical neglect (Viola et al., 2016). Furthermore, we observed significant sex differences among participants. Males reported higher levels of physical abuse and neglect than females. These findings are partially consistent with a previous study conducted in Italy, which found that males are more frequently exposed to physical abuse, whereas females are more frequently exposed to sexual and emotional abuse (Prino et al., 2018). Additionally, meta-analytic findings showed no sex differences for physical and emotional neglect (Stoltenborgh et al., 2013). In contrast, females reported higher levels of dissociation, especially cognitive/behavioral reexperiencing, supporting previous research showing that females may exhibit greater severity of complex posttraumatic stress symptoms, for which the dissociative domain of reexperiencing is a critical symptom cluster (Ho et al., 2021). In line with previous research (Orth et al., 2010), females also reported higher levels of shame.

Significant associations were found between other sociodemographic characteristics and the variables of interest. Specifically, negative associations were found between dissociative symptoms (with the exception of sensory misperception) and age, as well as between dissociative symptoms and years of education, in accordance with some studies (Espirito Santo & Luís Pio-Abreu, 2008; Maaranen et al., 2008). Also, negative associations were found between all forms of shame and age, and between overall and characterological shame and years of education. As the mean age of participants was 31.31 years, these findings partially support previous research suggesting that levels of shame may decline from adolescence to middle adulthood, regardless of educational level (Orth et al., 2010).

Correlation analyses showed that all types of child abuse and neglect were associated with a heightened severity of dissociative symptoms (including depersonalization/derealization, gaps in awareness and memories, sensory misperception, and cognitive/behavioral reexperiencing). These findings are consistent with previous research suggesting that childhood traumatic experiences are linked to increased levels of dissociation (Daniels et al., 2024; Vonderlin et al., 2018). In fact, child abuse and neglect may lead to persistent dissociative processes that serve to split off overwhelming trauma-related mental states from awareness and, thus, to paradoxically preserve internal coherence and prevent a sense of psychic fragmentation. However, chronic dissociative processes in childhood trauma survivors hinder the integration of mental states (Schimmenti & Caretti, 2016), resulting in multiple discontinuities in consciousness. In such circumstances, dissociative symptoms may reflect not only the disorganizing effects of childhood trauma on the psychic functioning or specific alterations in states of consciousness (Farina et al., 2019; Lanius, 2015), but also the persistence of dissociative processes in maintaining the compartmentalization of trauma-related mental states (Schimmenti, 2023).

Additionally, our results revealed positive associations between all types of childhood traumatic experiences and shame (including characterological, behavioral, and bodily shame), except for a non-significant association between physical neglect and bodily shame. Although previous research suggest that various types of childhood traumatic experiences are related to heightened shame (Kealy et al., 2023; Keene & Epps, 2016; Kim et al., 2009; Shahar et al., 2015), a recent meta-analysis found that shame is significantly associated with overall childhood traumatic experiences, emotional abuse, sexual abuse, physical neglect, and emotional neglect, but not with physical abuse (H. Zhang et al., 2025). From a developmental perspective, repeated failures in early attachment relationships, manifested as child abuse and neglect, undermine the development of self-representations as lovable and worthy, while also impairing the ability to regulate shame (Schore, 1998). Thus, survivors of childhood trauma may not experience shame as a transient emotional state, but rather as a persistent and relatively unprocessed emotion that deeply shapes their sense of identity (Benau, 2017).

In line with a recent meta-analysis showing a moderate positive association between dissociation and shame (Rudy et al., 2022), correlation analyses also revealed positive associations between all dissociative symptoms and shame (including characterological, behavioral, and bodily shame). It is noteworthy that the causal relationship between shame and dissociation is not fully understood. It has been proposed that shame may elicit dissociative experiences and, in turn, dissociative experiences may evoke shame, within a reciprocal and reinforcing relationship (Dorahy et al., 2017, 2021, 2025; Kouri et al., 2023). However, recent theoretical advancements in psychological trauma posited a dynamic relationship between dissociation and shame among individuals exposed to childhood trauma, suggesting that discontinuities in self-representation resulting from the compartmentalization of trauma-related mental states might foster feelings of shame (Schimmenti, 2022). From this perspective, we tested the potential mediating role of dissociation in the relationships between childhood trauma and shame.

SEM analysis showed that childhood trauma was a significant predictor of both dissociation and shame, and that the effect of childhood trauma on shame was partially mediated by dissociation. These findings may enhance the understanding of dissociation as an explaining mechanism of the relationship between childhood trauma and shame. From a psychodynamic perspective, shame-laden representations embedded in childhood traumatization may lead to the unconscious reenactment of self-devaluation and self-blame as an unconscious strategy of attributing meaning to one’s disturbing inner experience without directly confronting overwhelming trauma-related mental states. Accordingly, the dissociative compartmentalization of trauma-related mental states prevents psychic fragmentation but also reinforces feelings of shame among childhood trauma survivors (R. Chefetz, 2022; Schimmenti, 2022). Also, SEM analysis further supports the role of certain sociodemographic variables in increased levels of dissociation and shame. In fact, being female was a predictor of increased levels of dissociation and shame; younger age was a predictor of increased levels of dissociation and shame; and years of education were a predictor of decreased levels of dissociation.

The current study is not without limitations. First, the sample included participants from the general population. Therefore, the findings may not be directly generalizable to other populations, such as clinical samples. Moreover, participants were recruited through snowball sampling, which primarily relied on announcements disseminated via social media platforms. This recruitment method may have limited the representativeness of the sample. For instance, advertisements on social media may have increased the likelihood of recruiting individuals interested in the research topic (Lehdonvirta et al., 2021). It is also noteworthy that the sample was predominantly female (62.8%). Although the SEM model included sociodemographic characteristics as covariates, specifically sex, age, and years of education, the high proportion of female participants may have significantly influenced the results. In light of these limitations, future research should employ alternative sampling methods, such as probabilistic sampling techniques, to enhance the representativeness of the sample with respect to the general Italian population and to ensure a more balanced distribution of sociodemographic characteristics. Also, further research could examine the role of dissociation in the relationship between childhood trauma and shame in participants with dissociative disorders and other clinical conditions where shame represents a core symptom (e.g., avoidant personality disorder). Although the variables of interest were assessed using well-validated self-report instruments, it is acknowledged that self-report assessments carry an increased risk of bias. Future studies might evaluate these variables through structured and semi-structured interviews. It is noteworthy that the current study tested theoretically driven hypotheses concerning the relationships between childhood trauma, dissociation, and shame. These hypotheses acknowledge the potential role of dissociative symptoms in fostering feelings of shame, whereas it is unlikely that shame generates clinical symptoms such as depersonalization or amnesic compartmentalization. However, the current study relied on a cross-sectional design, which does not allow for the ascertainment of causal effects of one variable on another. Thus, longitudinal studies could be highly valuable in ascertaining the direction of these associations and the potentially reciprocal relationships between dissociation and shame. Finally, future research could investigate the role of additional potential mediating variables (e.g., early maladaptive schemas, difficulties in mentalizing, and emotion dysregulation) that might further explain the relationships between childhood trauma and shame.

## 5. Conclusions

The current study showed that dissociation partially mediated the positive association between childhood trauma and shame. Its limitations notwithstanding, our findings suggest that trauma-related dissociation may reinforce negative self-representations and, consequently, foster shame, which may manifest as a tendency toward self-devaluation and self-blame. The current study has critical implications for clinical interventions. Therapeutic approaches with traumatized individuals should address not only the overt symptoms of shame but also the underlying dissociative processes that potentially perpetuate their symptoms. Specifically, clinicians might help patients become aware of the circumstances in which feelings of shame emerge and their negative effects on individual functioning, while also promoting adequate emotion regulation strategies. It may be critical to adopt a sensitive approach toward patients’ disclosure of feelings of shame, in order to avoid intensifying their sense of self-devaluation (Kilborne, 1999) and to enhance their sense of safety within the therapeutic relationship (Bowlby, 1988). This may gradually facilitate the exploration of the developmental roots of patients’ negative self-representations and, consequently, support their revision. In this respect, recognizing dissociation as a central mediator of the relationship between childhood maltreatment and shame allows for more targeted therapeutic strategies aimed at fostering integration, self-cohesion, and ultimately, the restoration of a more compassionate self-narrative.

## Figures and Tables

**Table 1:** Descriptive statistics and sex differences.

	Full Sample					Males		Females				
	(N = 763)					(n = 284)		(n = 479)				
	M	(SD)	Range	Skewness	Kurtosis	M	(SD)	M	(SD)	*t*_(761)_	*p*	*d*
Age	31.31	(13.29)	18–65	1.06	−0.29	32.12	13.71	30.82	13.03	1.30	0.193	0.10
Years of education	14.35	(2.77)	5–21	0.14	0.71	14.31	2.93	14.38	2.67	−0.34	0.738	−0.03
CTQ-SF—Emotional abuse	7.97	(4.11)	5–25	1.65	2.17	8.00	4.09	7.95	4.13	0.15	0.881	0.01
CTQ-SF—Physical abuse	6.01	(2.59)	5–25	3.92	17.47	6.46	3.15	5.74	2.16	3.76	<0.001	0.28
CTQ-SF—Sexual abuse	6.13	(3.10)	5–24	3.40	11.88	5.97	2.82	6.23	3.25	−1.11	0.266	−0.08
CTQ-SF—Emotional neglect	10.03	(4.85)	5–25	0.91	−0.02	10.42	4.89	9.80	4.82	1.71	0.087	0.13
CTQ-SF—Physical neglect	6.60	(2.56)	5–20	2.08	4.40	7.08	3.03	6.31	2.18	4.05	<0.001	0.30
DSS—Depersonalization/derealization	0.66	(0.85)	0–4	1.55	1.86	0.59	0.79	0.71	0.88	−1.82	0.069	−0.14
DSS—Sensory misperceptions	0.47	(0.73)	0–4	1.95	3.66	0.46	0.73	0.47	0.72	−0.27	0.790	−0.02
DSS—Cognitive/behavioral reexperiencing	1.15	(1.08)	0–4	0.77	−0.46	0.98	1.05	1.25	1.09	−3.41	0.001	−0.26
DSS—Gaps in awareness and memory	1.15	(1.04)	0–4	0.75	−0.39	1.08	1.01	1.19	1.05	−1.42	0.155	−0.11
DSS—Total scale	16.68	(16.11)	0–80	1.19	0.90	15.16	15.58	17.58	16.37	−2.01	0.045	−0.15
ESS—Characterological shame	24.42	(9.22)	12–48	0.62	−0.55	23.08	8.96	25.21	9.30	−3.10	0.002	−0.23
ESS—Behavioral shame	20.46	(6.73)	9–36	0.29	−0.71	18.85	6.55	21.42	6.66	−5.18	<0.001	−0.39
ESS—Bodily shame	9.33	(3.84)	4–16	0.18	−1.18	8.06	3.59	10.08	3.79	−7.26	<0.001	−0.54
ESS—Total scale	54.21	(17.83)	25–100	0.41	−0.68	49.99	17.28	56.71	17.70	−5.11	<0.001	−0.38

Note: CTQ-SF = Childhood Trauma Questionnaire-Short Form; DSS = Dissociative Symptoms Scale; ESS = Experience of Shame Scale.

**Table 2:** Pearson’s r correlations among the investigated variables.

	2.	3.	4.	5.	6.	7.	8.	9.	10.	11.	12.	13.	14.	15.	16.
1. Age	0.20 ***	−0.13 ***	−0.05	−0.03	0.02	0.09 *	−0.13 ***	−0.06	−0.22 ***	−0.26 ***	−0.20 ***	−0.31 ***	−0.14 ***	−0.22 ***	−0.26 ***
2. Years of education	-	−0.09 *	−0.03	0	−0.06	− 0.09 *	−0.15 ***	−0.20 ***	−0.19 ***	−0.19 ***	−0.21 ***	−0.10 **	−0.02	−0.07	−0.07 *
3. CTQ-SF—Emotional abuse		-	0.52 ***	0.36 ***	0.60 ***	0.43 ***	0.37 ***	0.28 ***	0.40 ***	0.34 ***	0.39 ***	0.34 ***	0.28 ***	0.27 ***	0.34 ***
4. CTQ-SF—Physical abuse			-	0.36 ***	0.36 ***	0.54 ***	0.29 ***	0.28 ***	0.26 ***	0.21 ***	0.29 ***	0.13 ***	0.12 **	0.10 **	0.13 ***
5. CTQ-SF—Sexual abuse				-	0.20 ***	0.30 ***	0.20 ***	0.23 ***	0.21 ***	0.20 ***	0.24 ***	0.17 ***	0.13 ***	0.15 ***	0.17 ***
6. CTQ-SF—Emotional neglect					-	0.59 ***	0.19 ***	0.14 ***	0.18 ***	0.14 ***	0.19 ***	0.19 ***	0.14 ***	0.14 ***	0.18 ***
7. CTQ-SF—Physical neglect						-	0.26 ***	0.31 ***	0.19 ***	0.17 ***	0.26 ***	0.12 **	0.08 *	0.06	0.11 **
8. DSS—Depersonalization/derealization							-	0.76 ***	0.75 ***	0.68 ***	0.91 ***	0.45 ***	0.36 ***	0.38 ***	0.46 ***
9. DSS—Sensory misperceptions								-	0.62 ***	0.67 ***	0.85 ***	0.31 ***	0.25 ***	0.22 ***	0.30 ***
10. DSS—Cognitive/behavioral reexperiencing									-	0.74 ***	0.88 ***	0.49 ***	0.41 ***	0.39 ***	0.49 ***
11. DSS—Gaps in awareness and memory										-	0.89 ***	0.45 ***	0.37 ***	0.34 ***	0.45 ***
12. DSS—Total scale											-	0.49 ***	0.40 ***	0.38 ***	0.49 ***
13. ESS—Characterological shame												-	0.78 ***	0.62 ***	0.95 ***
14. ESS—Behavioral shame													-	0.62 ***	0.91 ***
15. ESS—Bodily shame														-	0.77 ***
16. ESS—Total scale															-

Note: CTQ-SF = Childhood Trauma Questionnaire-Short Form; DSS = Dissociative Symptoms Scale; ESS = Experience of Shame Scale; * *p* < 0.05; ** *p* < 0.01; *** *p* < 0.001.

**Table 3:** Estimates of direct, indirect, and total effects of childhood trauma on shame.

	β	*p*	95% C.I.
Direct effect of childhood trauma on shame	0.11	0.022	0.016–0.209
Indirect effect via dissociation	0.20	<0.001	0.142–0.253
Total effect of childhood trauma on shame	0.31	<0.001	0.230–0.391

## Data Availability

The data presented in this study are available on request. The data are not publicly available due to GDPR 2016/79.
